# Conditional inference trees in the assessment of tree mortality rates in the transitional mixed forests of Atlantic Canada

**DOI:** 10.1371/journal.pone.0250991

**Published:** 2021-06-18

**Authors:** Huiwen Guan, Xibin Dong, Guohua Yan, Tyler Searls, Charles P. -A. Bourque, Fan-Rui Meng

**Affiliations:** 1 College of Economics & Management, Zhejiang University of Water Resources and Electric Power, Hangzhou, Zhejiang, China; 2 Faculty of Forestry and Environmental Management, University of New Brunswick, Fredericton, New Brunswick, Canada; 3 College of Engineering and Technology, Northeast Forestry University, Harbin, Heilongjiang, China; 4 Department of Mathematics and Statistics, University of New Brunswick, Fredericton, New Brunswick, Canada; Technical University in Zvolen, SLOVAKIA

## Abstract

Long-term predictions of forest dynamics, including forecasts of tree growth and mortality, are central to sustainable forest-management planning. Although often difficult to evaluate, tree mortality rates under different abiotic and biotic conditions are vital in defining the long-term dynamics of forest ecosystems. In this study, we have modeled tree mortality rates using conditional inference trees (CTREE) and multi-year permanent sample plot data sourced from an inventory with coverage of New Brunswick (NB), Canada. The final CTREE mortality model was based on four tree- and three stand-level terms together with two climatic terms. The correlation coefficient (R^2^) between observed and predicted mortality rates was 0.67. High cumulative annual growing degree-days (GDD) was found to lead to increased mortality in 18 tree species, including *Betula papyrifera*, *Picea mariana*, *Acer saccharum*, and *Larix laricina*. In another ten species, including *Abies balsamea*, *Tsuga canadensis*, *Fraxinus americana*, and *Fagus grandifolia*, mortality rates tended to be higher in areas with high incident solar radiation. High amounts of precipitation in NB’s humid maritime climate were also found to contribute to heightened tree mortality. The relationship between high GDD, solar radiation, and high mortality rates was particularly strong when precipitation was also low. This would suggest that although excessive soil water can contribute to heightened tree mortality by reducing the supply of air to the roots, occasional drought in NB can also contribute to increased mortality events. These results would have significant implications when considered alongside regional climate projections which generally entail both components of warming and increased precipitation.

## Introduction

Long-term predictions of forest dynamics, including growth and species composition, are central to making sustainable forest-management decisions [1, 2]. Estimations of tree mortality rates are particularly important in developing long-term predictions of forest dynamics as mortality not only influences total growing stock, but also affects stand structure [3], floristic composition [3], as well as nutrient and carbon cycling [4]. Despite their importance, reliable estimations of tree mortality rates under different biotic and abiotic site conditions are often difficult to obtain. This is because tree mortality is one of the least understood processes in forest ecosystems due to the complex, multi-scale interactions between growing-environment variables, as well as the influence of biotic and abiotic site factors [5]. Studies have attempted to establish connections between mortality and external factors, such as fire [6, 7], insect defoliation [8], and climatic variability [9, 10], but attempts to quantify internal sources of tree mortality are limited. These internal factors, including species identity [10], diameter at breast height [11], basal area [12], tree and stand age [13, 14], diameter growth rate [15], and stand competition [16–20] are known to each influence a tree’s likelihood of mortality, although to what degree is frequently unclear.

Many models have been developed to estimate tree mortality probability distributions in accordance with tree, stand, and environmental factors [15]. Logistic regression is broadly used to model tree mortality rates and their inverse functions, tree survival rates [6, 15, 21–23]. The Kaplan-Meier method, which is a non-parametric method [10], as well as artificial intelligence have both been used to model tree mortality [24]. However, the transformation of modeled results from a probability distribution to meaningful tree mortality rates is seldom straightforward. Further still, most results tend to focus on tree-level, binary predictions of “dead or alive”; distributed randomly with a variance that is in accordance with tree characteristics [25]. Such predictions are most suitable for more simplistic stand structures [25, 26]. Where mixed forests are concerned, the representation provided by these methods becomes overly complex [6, 27]. The development of alternative methods to estimate tree mortality rates is, as a result, an active and much needed research area in forestry.

Classification and regression trees (CART) are an implementation of recursive partitioning, which has been applied across a diversity of fields, including data mining, wastewater treatment studies, and estimating landslide susceptibility [28–31]. Tree-structured models have the advantage that modeled results can be visually interpretable in the assessment of tree mortality rates. However, CART is susceptible to overfitting and selection biases, which favors covariates with more potential splits [28]. Conditional inference trees (CTREE) resolve the overfitting and selection bias problems associated with CART by applying suitable statistical tests to variable selection strategies and split-stopping criterion [32, 33]. The CTREE method has been employed in various contexts, such as in the testing of automobile engines [34] and in the characterization of myocardial infarctions [35, 36].

In this study, we use CTREE to model tree mortality rates for major commercial tree species in New Brunswick, Canada, using long-term tree data sourced from the provincial permanent sample plot (PSP) inventory [37]. We have also analyzed the impacts of environmental factors on tree mortality rates by species, giving particular attention to the influence of climatic variables, including precipitation and temperature.

## Materials and methods

### Study area

**Fig 1:**
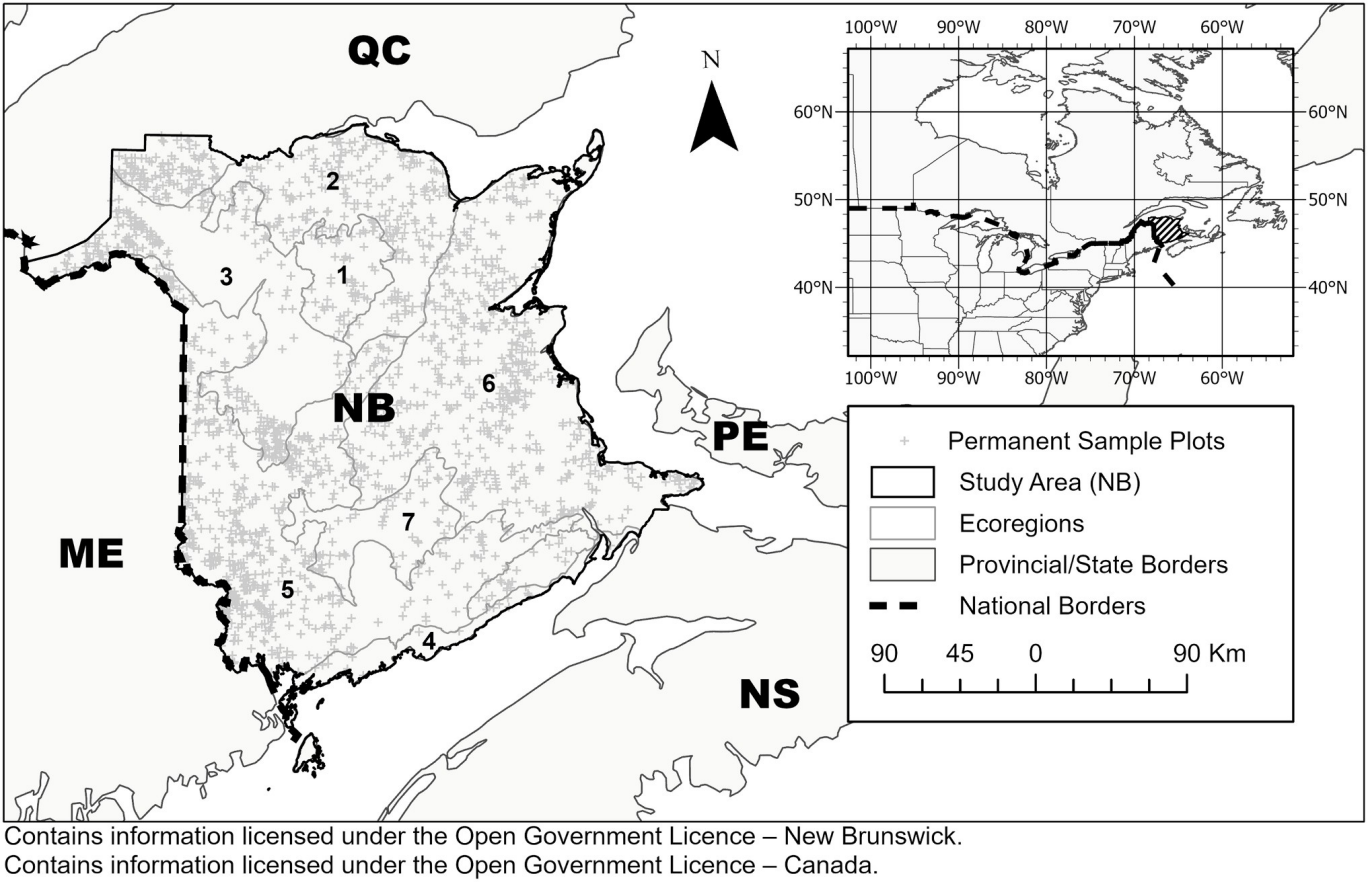
Location of permanent sample plots and ecoregions in New Brunswick of eastern Canada (inset). Ecoregion number in the legend corresponds to that in Table 1.

The study area for this research is the province of New Brunswick (NB), Canada (Fig 1). Forested area makes up more than 85% of the NB’s seven million hectares (ha). The province is comprised of seven distinct ecoregions [38] (Table 1, Fig 1), classified based on prevalent climatic, geologic, topographic, and floristic communities and conditions [39]. NB’s climate has characteristic cold, snowy winters, and warm, humid summers with an annual average temperature range from 2.0–6.3°C. Mean annual precipitation ranges from 1,000–1,500 mm, with about half occurring as snow [40].

**Table 1:** Ecoregion and permanent sample plot (PSP) inventory summary, where A_r_ is the ecoregion land area (km^2^), A_rp_ the proportion of NB that makes up the ecoregion (%), C_r_ the number of PSPs within the ecoregion, and C_rp_ the proportion of plots which constitute NB’s total PSP inventory within a specific ecoregion (%).

Ecoregion No.	Ecoregion	Area		Plots	
		A_r_	A_rp_	C_r_	C_rp_
1	Highlands	4,908	6.74	421	11.65
2	Northern Uplands	8,761	12.03	577	15.97
3	Central Uplands	12,008	16.49	527	14.59
4	Fundy Coastal	2,223	3.05	39	1.08
5	Valley Lowlands	20,278	27.85	903	24.99
6	Eastern Lowlands	20,856	28.64	1,047	28.98
7	Grand Lake Lowlands	3,779	5.19	99	2.74
	**Total**	**72,813**		**3,613**	

New Brunswick is home to 39 native tree species, the majority of which are conifers. Of these native species, 28 occur within the provinces permanent sample plot (PSP) inventory (Table 2). Commercially significant conifers include balsam fir (*Abies balsamea*), red spruce (*Picea rubens*), white spruce (*Picea glauca*), and black spruce (*Picea mariana*). Abundant shade intolerant hardwoods include trembling aspen (*Populus tremuloides*), red maple (*Acer rubrum*), and white birch (*Betula papyrifera*), whereas shade tolerant hardwoods include yellow birch (*Betula alleghaniensis*), sugar maple (*Acer saccharum*), ironwood (*Ostrya virginiana*), black cherry (*Prunus serotina*), white ash (*Fraxinus americana*), red oak (*Quercus sp*.), and American beech (*Fagus grandifolia*) [41].

**Table 2:** Tree species common and scientific names, where N is the number of tree mortality observations.

Common Name	Scientific Name	Code	N
American Mountain Ash	*Sorbus americana*	AA	305
Black Ash	*Fraxinus nigra*	AB	607
White Ash	*Fraxinus americana*	AW	1,294
Grey Birch	*Betula populifolia*	BG	3,751
White Birch	*Betula papyrifera*	BW	33,489
Yellow Birch	*Betula alleghaniensis*	BY	13,040
Pin Cherry	*Prunus pensylvanica*	CP	1,950
Eastern White Cedar	*Thuja occidentalis*	EC	16,816
American Beech	*Fagus grandifolia*	EE	15,874
Eastern Hemlock	*Tsuga canadensis*	EH	1,418
Balsam Fir	*Abies balsamea*	FB	163,119
Hoptree	*Ptelea trifoliata*	IW	878
Speckled Alder	*Alnus incana*	KA	436
Tamarack	*Larix laricina*	LT	3,739
Mountain Maple	*Acer spicatum*	MM	1,868
Red Maple	*Acer rubrum*	MR	56,676
Sugar Maple	*Acer saccharum*	MS	24,358
Striped Maple	*Acer pensylvanicum*	MT	5,436
Red Oak	*Quercus rubra*	OR	267
Jack Pine	*Pinus banksiana*	PJ	8,142
Red Pine	*Pinus resinosa*	PR	448
Balsam Poplar	*Populus balsamifera*	RB	776
Large-tooth Aspen	*Populus grandidentata*	RL	1,561
Trembling Aspen	*Populus tremuloides*	RT	21,137
Black Spruce	*Picea mariana*	SB	108,378
Red Spruce	*Picea rubens*	SR	62,614
White Spruce	*Picea glauca*	SW	21,158
Willow	*Salix nigra*	XW	257

### Permanent sample plot data

NB’s permanent sample plot (PSP) inventory is maintained by the NB Department of Energy and Resources Development. The network containing nearly 1,900 PSPs, more than 165,000 individual trees, and 580,000 tree measurements spanning a period from 1985–2014 [37]. Plots in NB use a standard area of 400 m^2^ and are re-measured at periods of 3 or 5 years depending on stand age [37]. The spatial distribution of NB’s network of PSPs is illustrated in Fig 1. Tree species and diameter at breast height (DBH) are recorded by technicians for every live tree in the plot with a DBH > 5.1 cm. All tree records from PSPs that had been subject to fire, insect defoliation, windthrow, or timber harvesting were excluded from the analysis. The modeling methods employed cannot account for the stochastic nature of large-scale mortality (or ‘calamity’) events.

In NB’s PSP inventory, tree age is estimated within plots using the measured age of sample trees located immediately outside plots; this minimizes the potential of injuring trees within the plot through the increment coring process. Sample tree age is then used to inform age class(es) present within a plot. All stems within the plot are then assigned an age class by technicians undertaking plot measurements. Age is only measured once within a plot’s lifecycle; observed as part of the plot establishment record. A collection of approximately 27,500 sample age measurements informs all tree age measurements in NB’s PSP inventory.

### Model development

#### Data quality control and variable selection

A complete list of independent variables is provided in Table 3. Tree species were coded as a nominal variable, with 28 species levels (Table 2).

**Table 3:** Listing of independent variables for tree rate of mortality modeling.

Variable	Explanation	Class
ΔBA	Average annual basal area growth increment between two plot measurements (cm^2^ yr^-1^)	Tree-level
SP	Species	Tree-level
AGE	Age class	Tree-level
BAL	Total basal area of all trees with diameter > diameter of the subject tree (m^2^ ha^-1^)	Tree-level
GDD	Growing degree-days (degree-day units)	Climatic
PCP	Precipitation (mm)	Climatic
INS	Potential solar radiation (Wh m^-2^)	Stand-level
SLP	Slope (%)	Stand-level
ERD	Relative density (%)	Stand-level

#### Tree-level variables

Tree age is often used as the basis for models of individual-tree rate of mortality [23, 42–44]. In this study, four age classes were used as independent levels constituting the nominal AGE covariate: young, immature, mature, and overmature. Tree growth rates are also commonly used to estimate the probability of tree survival [5, 45]. In this study, the average annual basal area growth increment for individual trees (*ΔBA*), as measured between two consecutive *BA* measurements, was also selected as an independent variable:

$${\Delta BA}_{i}=\frac{{BA}_{i}-{BA}_{i-1}}{t_{i}-t_{i-1}}$$ (1)

where *BA*_*i*_ is the tree basal area at the *i*^th^ measurement, and *t*_*i*_ is the corresponding year of measurement. Observations of *ΔBA* > 0.02 cm yr^-1^ were considered outliers and were removed from the study.

#### Competition

Trees in stands face competitive interactions [20, 46, 47]. We used two variables to capture the influence of competition on tree mortality, namely (i) the total basal area of stands for trees thicker than the subject tree (*BAL*_*i*_), and (ii) an extended relative density index (*ERD*). The *BAL*_*i*_ index reflects the relative advantage of the tree as compared to other trees in the plot, and was calculated as the sum of total basal area of all trees with DBH’s greater than that of the subject tree [23, 48], i.e.,

$${BAL}_{i}=(\sum_{j=1}^{m}{BA}_{j})(\frac{10000}{AP})$$ (2)

where *BAL*_*i*_ is the total basal area of trees greater than *i*^th^ tree (m^2^), *AP* the size area of the PSP (m^2^), *BA*_*j*_ the basal area of the *j*^th^ tree, and *m* the number of trees in the plot with DBH greater or equal to the DBH of the *i*^th^ tree. *ERD* was developed in this study as a ratio between stand density and the maximum potential density of the stand to further account for overall competition intensity within the stand. The maximum potential density of the stand was calculated based on the law of self-thinning [46]. In accordance with the law, the maximum number of trees a stand can support decreases exponentially with mean tree size, i.e.,

$$N_{max}=\alpha {\left(\frac{DBH}{{DBH}_{r}}\right)}^{-\beta }$$ (3)

where *N*_*max*_ is the maximum potential density of the stand with mean DBH. Parameter *α* is the maximum density at an arbitrary reference DBH_r_ (set at 20 cm for this study, [68, 69]), and *β* is a self-thinning coefficient. In this study, we assume *N*_*max*_ to be constant irrespective of stand composition and age. For a stand with density *N* (stems ha^-1^), the extended relative density is then giving as,

$$ERD=\frac{N}{N_{max}}=\frac{n}{\alpha }{\left(\frac{DBH}{{DBH}_{r}}\right)}^{\beta }$$ (4)

Because *ERD* and *BAL* are closely related, but represent different aspects of competition-induced tree mortality, one interaction covariate, *BAL×ERD* was used to accommodate competitive forces in the CTREE model. Observations of *BAL×ERD* > 7 were considered outliers and were removed from further consideration.

#### Stand-level climatic factors

Four stand-level climatic (i.e., abiotic) factors were included in the analysis, i.e., annual precipitation (PCP), potential solar radiation (INS), annual cumulative growing degree-days (GDD), and slope. Slope was included here as a stand-level factor, as it was anticipated to be a fair proxy of soil moisture conditions within a given PSP. Fine-scale soils information, such as depth, porosity, and frost-depths were unavailable for the study area. A percentage slope surface was calculated from a digital elevation model (DEM) interpolated at 1-m resolution [49]. Plot slope was then estimated as the average slope within a 40-m radius from the plot center. Plots where slope observations > 40% were considered outliers and were removed from the study. Potential solar radiation was calculated as the sum of direct and diffuse solar radiation. DEM-based INS was determined as a function of solar angle by latitude, slope, as well as aspect, and was evaluated as a raster surface [50]. As with the slope variable, plot estimations of INS (in Wh m^-2^) were calculated as the average within a 40-m radius from the plot center.

Utilizing the same methods leveraged in the JABOWA-family of forest gap models, GDD was estimated using only monthly mean daily temperatures [51]. In the estimation of GDD, the base temperature below which tree growth is assumed to be negligible [51] was assumed to be 4.4°C for all species. Monthly mean daily temperatures at each PSP were determined through monthly mean daily maximum and minimum temperatures, as generated with ANUSPLIN, a non-parametric surface-fitting procedure [52]. The ANUSPLIN-generated datasets used herein were based on records obtained from weather stations and then interpolated over topographic surfaces. Through the same approach we also obtained estimates of cumulative monthly PCP, which we then used to determine cumulative annual PCP. The historical monthly models generated with ANUSPLIN have 95% confidence limits of approximately ±1.1 and ±1.3°C for maximum and minimum daily temperatures, respectively, and 10–40% for monthly PCP [52]. Both cumulative annual PCP and GDD were employed as the annual average between previous and current subject measurements.

A five-year tree rate of mortality during the period between two consecutive measurements served as the only dependent variable. Rate of mortality was estimated based on tracking individual trees over time and taking note of their mortality-status change. As a general practice, tree status was coded as “alive” or “dead” at the time of inventory measurement. In this study, living trees were coded as 1, and dead trees as 0. Repeated codes of 0 for the same tree were deleted following the earliest instance of code 0. This avoided repeated counting of dead trees. To calculate rate of mortality, all data were divided into groups according to species and other independent variables (Table 2), with an objective to minimize the within-group mortality rates. Those groups with insufficient records (< 20) were eliminated. After that, the five-year mortality rates were calculated based on the number of dead trees during the measurement period in the group and the total number of living trees at the beginning of the measurement period. For a remeasurement period of other than five years, the method introduced by Flewelling and Monserud [70] was used to convert 3-year mortality rates (P_m3_) to five-year rates by means of P_m5_ = 1-(1-P_m3_)^5/3^.

### Covariate testing

**Fig 2:**
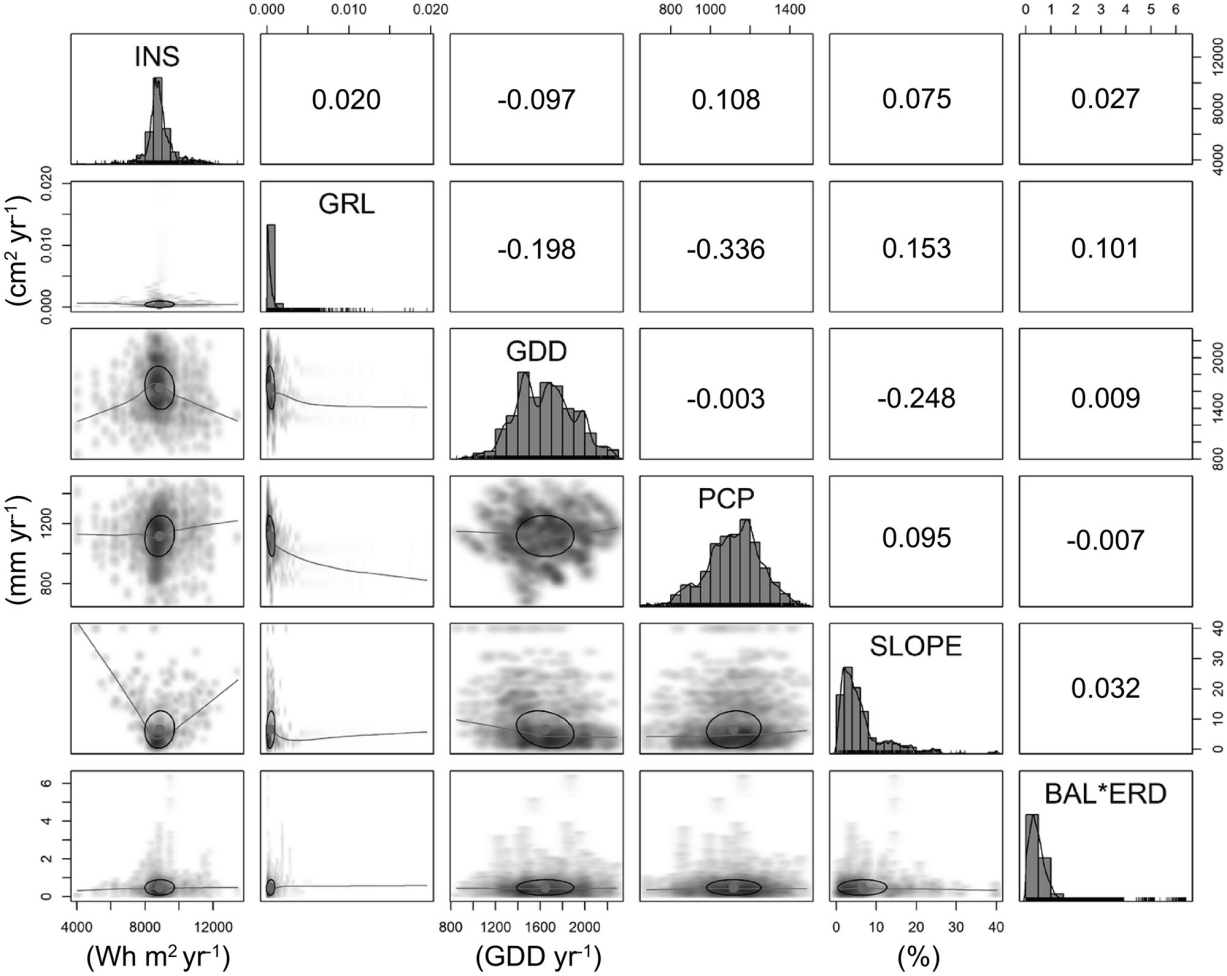
Correlation matrix for the nine independent variables used in the modeling methods. Variable distribution is shown along the diagonal, Spearman rank correlation between the corresponding independent variables in the upper right quadrant.

To test for multicollinearity, a correlation matrix was prepared using each of the nine covariates (Fig 2). Spearman rank correlation was used to determine correlation values [53]. Commonly, Spearman correlation coefficients between 0.10 and 0.29 represent a slight association, those between 0.30 and 0.49 represent some association, and those > 0.50 represent a significant association or relationship. We found most independent variables to have no correlation with one and other (Fig 2). Potential solar radiation (INS), however, correlated slightly with precipitation (PCP), as did growth increment (*ΔBA*) with *BAL×ERD*; *ΔBA* was also found to correlate slightly with slope (SLP). Correlation amongst model covariates was determined to not be of significant concern.

### Model structure

The CTREE mortality model was fitted using the “*partykit*” package in R [36, 54]. CTREE generates a nonlinear mortality diagram, which gives a tree-shape probability map of tree mortality rates. The initial bifurcation was determined through multiplicity-adjusted *p*-values, in accordance with Bonferroni’s criterion [36, 54]. CTREE is implemented through the following protocol [36, 54], i.e., at each stage, CTREE determines the optimal split of a region in the feature space assembled by variables that affect tree mortality and partitioned according to the Bonferroni criterion [36]. The splitting process starts by using the entire feature space and repeats, informing successive splits using the remaining feature space. This process continues with risk-tree development and pruning until the terminal node is realized and no subsequent splits are possible. The probability of a tree dying is calculated at each terminal node, which yields a stratified model.

### Model evaluation and comparison

Correlation coefficients between observed measurements and model predictions, as well as mean square error (MSE) and mean biases were used to assess the model. Furthermore, cross validation of CTREE model was completed through a k-fold test [55]. The original dataset was randomly partitioned into 5 subsamples (k = 5). One subsample (20% of the data in the original dataset) was chosen to serve as the test series for cross validation, and the remaining four subsamples (20% each) for model development (training). The cross-validation process was repeated 5 times, with each subsample being utilized once for training purposes. A two-tailed *p*-value < 0.05 was considered statistically significant.

## Results and discussion

**Fig 3:**
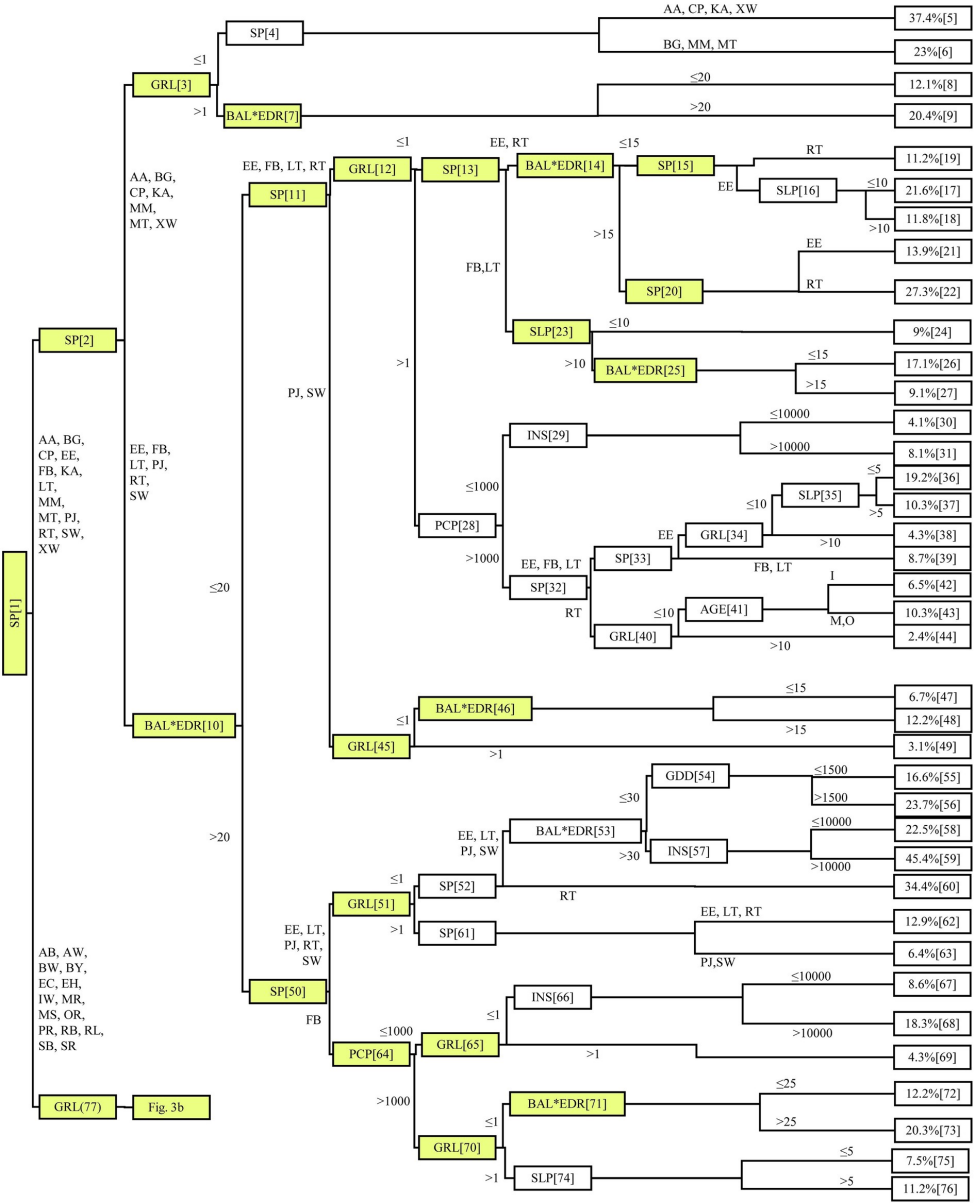
Nodes 1–76 of the conditional inference tree (CTREE) of rate of mortality for 28 boreal and temperate species (see Table 2, for code definition). Tree-level variables include species (SP), average annual basal area growth increment (*ΔBA*), basal area of the largest tree and relative density interaction (*BAL×EDR*). Stand-level variables include potential solar radiation (INS) and slope (SLP). Climatic variables include cumulative annual growing degree-days (GDD) and cumulative annual precipitation (PCP). The terminal nodes, 38 in total, show the proportion of dead trees. Nodes shaded yellow were consistent in all folds in model cross-validation.

**Fig 4:**
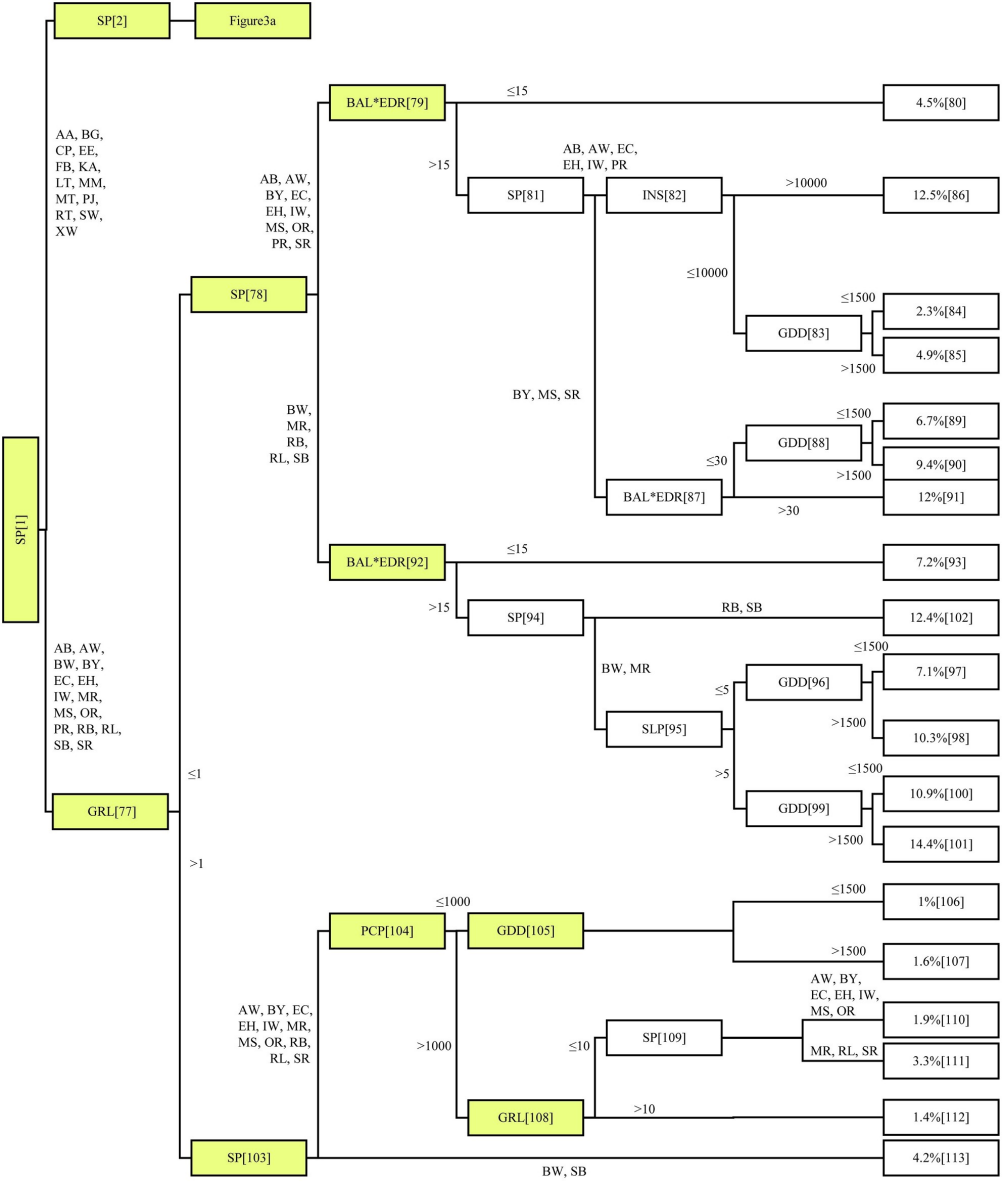
Nodes 77–108 of the conditional inference tree (CTREE) of rate of mortality for 28 boreal and temperate species (see Table 2, for code definition). Tree-level variables include species (SP), average annual basal area growth increment (*ΔBA*), basal area of the largest tree and relative density interaction (*BAL×EDR*). Stand-level variables include potential solar radiation (INS), and slope (SLP). Climatic variables include cumulative annual growing degree days (GDD) and cumulative annual precipitation (PCP). The terminal nodes, 19 in total, show the proportion of dead trees. Nodes shaded yellow were consistent in all folds in model cross-validation.

The correlation coefficient between observed and predicted mortality was 0.67, with a MSE of 0.006. Mean bias was observed to be < 0.001. The final mortality model had 56 inner nodes and 57 terminal nodes (Figs 3 and 4). For purposes of discussion, notation N36 refers to Node 36, N47 to Node 47, and so forth.

Through the k-fold test, correlation coefficients ranged from 0.59 to 0.62 with a mean of 0.61, and MSE ranged from 0.006 to 0.008, with mean of 0.007. The difference in correlation coefficients between each k-fold subset was < 0.03, suggesting that the accuracy of the CTREE procedure to be relatively stable, even with a reduced sample size. CTREE’s branching structure was affected by the reduced sample size used in the k-fold validation, i.e., the number of terminal nodes in the k-fold mortality models ranged from 43 to 52, and the number of inner nodes from 42 to 51. We observed that despite these structural differences, the higher-order splits from 1 to 4 of the CTREE did not change between k-fold subsets (Figs 3 and 4). Rather, most differences in split structure were found to occur amongst the lower branches of the probability tree. The split in Node 28 (the one associated with PCP) was not common to all data subsets. One instance was found to appear after the species split. Precipitation (PCP) was an important contributor to tree mortality in both probability tree structures, but the relationship to other covariates markedly differed. Such an observation confirms the significance of sample size in the fit of the probability tree. In more than one of the CTREE-models prepared as part of the k-fold testing, the splits at N4 and N7 were not present (Fig 3). This result identifies that the CTREE method can avoid issues of overfitting by eliminating frivolous nodes where the input dataset does not support additional branching before the terminal node. The CTREE method offers a mortality modeling approach where complex nonlinear relationships between covariates and mortality rates are determined through the CTREE model package. This is in contrast to many established mortality modeling approaches, where the nonlinear relationships between covariates and mortality rates depend on having a prior understanding and stipulating through model development [56, 57]. Frequently, the empirical study of such nonlinear relationships between variables in the growing environment and tree mortality is not available.

### Influence of tree-level variables on tree mortality

We found that the species covariate SP was the most important discriminator, appearing not only for the first split, but for subsequent splits as well (Figs 3 and 4). Red oak and eastern hemlock have the lowest rate of mortality range between 1.00–1.90% (N105, N108, and N109; Fig 4). Balsam fir was found to have significant variability in rate of mortality, ranging from 8.80% (N33) to 20.3% (N71; Fig 3). The high variability in balsam fir mortality rate is intuitive, given the species is short-lived and has a tendency to form high-density cover [58], whereas oak and hemlock are long-lived, shade tolerant hardwoods [59]. Annual growth rate was found to be the most important predictor of tree mortality [5, 45]. Studies have previously found tree mortality and growth to be inversely related [60, 61], and furthermore, that tree growth is continuous through life in uneven aged stands [62]. Low *ΔBA* was also found to lead to higher mortality rates (Fig 2a). Mortality was as high as 23–37.4% (moderated by species) whenever *ΔBA* ≤ 1 cm^2^ 5-yr^-1^, as compared to 12.1–20.4% (moderated by competition indices) when *ΔBA* > 1 cm^2^ 5-yr^-1^ (Fig 3). Similar results were observed through N12, N45, N51, N65, N70, and N77 (Figs 3 and 4). When *ΔBA* > 10 cm^2^ 5-yr^-1^, rate of mortality was generally reduced, e.g., a rate of mortality of 2.4% was observed when *ΔBA* > 10 cm^2^ 5-yr^-1^, but 8.4% when *ΔBA* ≤ 10 cm^2^ 5-yr^-1^ (i.e., N40; Fig 3).

The AGE covariate appeared only once in the CTREE mapping (i.e., N41; Fig 3); branching trembling aspen mortality rates. Aspen mortality rates were 6.5% and 10.3% for immature trees and mature/overmature trees, respectively. This split only occurred where aspen *ΔBA* < 10 cm^2^ 5-yr^-1^ through the preceding five years. When *ΔBA* > 10 cm^2^ 5-yr^-1^, aspen mortality rate was 2.4%, regardless of AGE class. Perhaps contrary to intuition, our results do not support age effects as a significant contributor to rate of mortality. This could be attributed to two causalities, (i) the forests in the study area are intensively managed with few trees reaching full maturity and succumbing to age-related mortality, and (ii) given the inclusion of *ΔBA* as an independent variable, age effects may be indirectly applied through that term, as overmature trees tend to stop growing before mortality occurs. Still, the correlation matrix (Fig 2) identified no collinearity between *ΔBA* and AGE.

### Influence of competition on tree mortality

The impacts of *BAL* appear to be absorbed by the interaction between *BAL* and *EDR*, i.e., greater interaction-values associated with higher mortality rates. As shown in Fig 3, through N10, the average rate of mortality was 10.85% when *BAL×EDR* ≤ 20, as compared to 17.52 when *BAL×EDR* >20 (*BAL* in cm^2^ and *EDR* in number of stems per ha). Similar results were observed with CTREE N14, N25, N46, N79, N53, N87, and N92 (Figs 3 and 4). Variables representing competitive interactions were associated with higher mortality rates. As the two variables were used to reflect different competitive concerns, we found an interaction term to offer the best means to include the two otherwise intimately related parameters. Due to the generalized significance of this interaction to tree mortality, we can conclude that competition is one of the important factors causing death in trees. Indeed, studies have found that the majority of trees die young due to competitive pressures [63]. Other studies have found mortality rates to increase with increased water deficits and stand basal area [64].

### Influence of climate and site conditions on tree mortality

We found PCP to appear three times in N28, N64, and N104, and again in N35, N74, and N95 for SLP (Figs 3 and 4). High tree mortality rates were often coincidental with PCP < 1000 mm yr^-1^ (Figs 3 and 4). Through N28 (Fig 3), mortality rates for American beech, balsam fir, tamarack, and trembling aspen were 4.1–8.1% when PCP was < 1000 mm yr^-1^, and 2.4–19.2% when > 1000 mm yr^-1^. The mean slope (SLP) appeared to have a synergistic relationship with PCP, as observed in N35, where the rate of mortality for American beech was 19.2% when PCP > 1000 mm yr^-1^ and SLP < 5%, as compared to 10.3% when SLP > 5%. Still, SLP < 5% was not always associated with lower rate of mortality. Through N99 (Fig 4), SLP < 5% was associated with a lower white birch and red maple mortality rate (7.1–10.3%) as compared to 10.9–14.4% when SLP was > 5%. A similar trend was observed for balsam fir (N74), with a mortality rate of 7.5% with SLP > 5%, compared to 11.2% with SLP < 5%. In general, the impact of PCP and SLP were species dependent, and often synergistic with other independent terms in the CTREE model.

Solar radiation (INS) was found to increase the rate of mortality in four CTREE splits (N29, N57, N66 and N82; Figs 3 and 4). Through N29, mortality rates in four tree species (i.e., American beech, balsam fir, tamarack, and trembling aspen) were 4.1% when INS ≤ 10000 Wh m^-2^ and 8.1% when INS > 10000 Wh m^-2^. As INS has a warming effect on microclimate, in addition to direct impacts on photosynthetic potential, there may have been some overlap between GDD and INS. In this study, INS did not consider the year-to-year variations in cloud cover and associated reductions in available sunlight. Furthermore, our correlation matrix did not identify significant collinearity between the independent terms (Fig 2). We found the relationship between GDD and tree mortality to be mixed, splitting at six locations (N54, N83, N88, N96, N99, and N105), and involving 18 special interactions (Figs 3 and 4). In all cases, GDD > 1500 was associated with increased rate of mortality. Through N96, as an example, rate of mortality for white birch and red maple was 7.1% at < 1500 and 10.3% at > 1500 degree-days. Essentially, this characterization would suggest that a mean increase in regional temperatures, as is often attributed to changing climate, could lead to greater mortality in some tree species in NB.

PCP < 1000 mm yr^-1^ and GDD > 1500, possibly indicative of localized drought, contributed to a high rate of mortality (N106 *vs*. N107; Fig 4). This indicates that although excessive soil water content can lead to high mortality rates (i.e., SLP < 5%, PCP > 1000 mm yr^-1^, N36; Fig 3), occasional drought can also lead to high tree mortality rates, in agreement with other researchers’ findings [65]. For example, Peng et al. [65] found that drought conditions can induce an increase in tree mortality rate in old-growth forests. In general, our results identify that climatic variables, including GDD, cumulative annual PCP, and INS had significant impact on tree mortality rates for some tree species. There is a wealth of scientific literature that support the significance of climate to tree growth [9, 66, 67].

CTREE offers a viable method for estimating mortality rates in mixedwood, boreal-temperate ecotone making up the New England-Acadian forest. This study included nine independent variables within the CTREE model, none of which have been determined to be significantly collinear with one and another (Fig 2). Through the design phases of this study program, we undertook various tests to explore the value of an array of potential covariates, eliminating those which did not offer a significant contribution to the final mortality model. While we recognize that certain covariates may have a lesser influence on tree mortality rate (e.g., *BAL×EDR* and INS), the inclusion of these terms improved the overall explanatory power of the model in our analysis. If either term (i.e., *BAL×EDR* and INS) was removed from the final CTREE model, the correlation coefficients would have been reduced from 0.671 to 0.597 or to 0.659, respectively. The decision to include the nine variables we ultimately chose was governed in large part by the breadth of the datasets available. While we recognize that tree mortality is an incredibly complex process, which is influenced by an exhaustive array of forest variables and interactions, the modeling methods employed in this study do not attempt to illuminate the influence of stochastic [68, 69] or periodic [70] disturbance on tree mortality rates. The influence of these type of disturbances on mortality would need to be considered alongside our CTREE model in any practical application of the methods.

## Conclusions

Perhaps intuitively, our CTREE approach to mortality confirmed rate of tree growth and competitive interactions at the stand-level as important determinants of tree mortality rate. We found GDD and INS to increase mortality for 18 species, including white birch, black spruce, sugar maple, and tamarack. We also found that high PCP and shallow SLP commonly contributed to and increased tree mortality rates, presumably as a result of excess soil water in some parts of NB. We observed that low PCP in combination with high GDD or high INS often led to elevated tree mortality, potentially indicative of the effects of drought. These observations may have significant implications when considered alongside regional climate projections for NB, which generally entail both components of warming and increased precipitation. The major contribution offered by the CTREE approach is the expanded capacity to reveal complex nonlinear relationships in mortality, without the relationships needing to be known *a priori*, as is commonly the case in tree mortality modeling. There is potential that the CTREE method may offer an improved means to modeling tree mortality in complex forested ecosystems, such as the New England-Acadian forest ecotone. In addition, the CTREE model could be directly integrated with forest growth and yield models, further bolstering the approach’s operational potential.
